# Editorial Correspondence

**Published:** 1877-08

**Authors:** W. C. Blalock

**Affiliations:** Barnesville, Ga.


### EDITORIAL CORRESPONDENCE.

BARNESVILLE, GA., August 15, 1877.

*Dr. John G. Westmoreland:*

DEAR DOCTOR—I have just dismissed one of the most interesting and remarkable cases it has ever been my lot to have in charge, and if you will not think me tiresome, will try to give you a succinct history of it. Should you think it worthy of laying before your readers, you are at liberty to do so.

I was called on June 15th to attend Mrs. M., and found a case of labor at full term, which was terminated without any untoward event in two hours after my arrival. Her lying-in was attended with no unfavorable symptoms, and in eight days she was up, and, to all appearances, doing well. I saw her no more until the morning of June 29th, when I was summoned hurriedly to her bedside. I found her perfectly unconscious; pupils dilated; pulse 110. While making some inquiries as to her previous condition, I first noticed a slight twitching of the muscles of right side of face and arm. I suspected at once what was approaching, and quicker than the time it requires to tell it, a convulsion was upon her, with all its horrors. I realized the situation at once, and proceeded to act according to my best judgment. I proceeded to bleed her copiously, and with the constant use of chloroform by inhalation, I succeeded in arresting the convulsions about the middle of the afternoon.

I remained by her bedside during the night, looking each hour for the end to come. She, however, rallied during the latter part of the night; and without any sign of returning convulsions, on the morning of the 30th, while making a close examination of her case, I found her with complete paralysis of the right side, with loss of voice. I found considerable tenderness over the kidneys, with urine scanty. I diagnosed her case as one of urmæic poisoning, and proceeded to treat her accordingly. Gave her chloroform internally, with an occasional dose of squills and spirit nitre; expecting by the former to stimulate the cerebrum, and also to destroy the urea in the blood. The latter was given with a view of stimulating the kidneys to action. I had early in the morning given a brisk cathartic and applied a blister to nape of neck, the therapeutical indications being to promote the excretion of the urea by the proper emunctories, and also to favor its vicarious elimination by the bowels.

I will now give you a statement of her case, as taken from day to-day:

July 1st. Resting quiet, having passed an ordinary night; pulse 120; tongue slightly coated; complete paralysis of right side; slight coma. Still continued the chloroform internally, in twenty-drop doses every three hours, and occasionally a tablespoonful of the spirit nitre and syrup of squills.

July 2d. No improvement; continued treatment, with the exception of dropping off the squills and nitre, and giving iustead, small doses of quinine.

July 3d. I think Mrs. M. better to-day; she passed a quiet night; no fever; pulse 86; kidneys secreting finely. No improvement in paralysis; continued treatment of chloroform aud nitre; applied blister over each kidney, as there was considerable tenderness.

July 4th. Condition not improved; put her upon strychnine and quinine, leaving off everything else.

July 5th. No improvement in Mrs. M. to-day. Began use of electro-magnetism; continued treatment; some fever to-day.

July 6th. Passed a restless night; decidedly worse; slight twitching of muscles of affected side. Put her upon chloroform inhalations and twenty-drop dose internally every three hours.

July 7th. No improvement in her condition to-day. Was compelled to administer the inhalation of chloroform through entire night; convulsions imminent.

July 8th. Mrs. M. passed a quiet night under morphine and quinine. No sign of convulsions this morning; kidneys torpid; no improvement in paralysis or speech.

July 9th. Mrs. M.'s condition not much improved. Put her to-day upon iron, strychnine, and calisaya bark, with the use of electro-magnetism.

July 10th. About as yesterday; paralysis and loss of voice still prominent. Continued treatment.

July 11th. Appetite good to-day for first time; no fever; looking brighter; yet no improvement in paralysis; no return of voice. Continued treatment.

July 12th. Passed a good night, with symptoms about as yesterday.

July 13th. Had a slight fever last night; did not rest so well. Shall put her on small doses quinine, with morphine. Kidneys doing very well; bowels regular and natural.

July 14th. Slight improvement on yesterday. Began the use of iron, strychnine, and calisaya bark again.

15th. Was called to see Mrs. M. this morning; found her in convulsions. Had several during the night. I arrested them with chloroform inhalations. Pulse weak, and 125 per minute; pupils dilated; surface bathed in cold perspiration. Succeeded in reviving her by friction and mustard to extremities. I had about exhausted my skill, and soon saw that she must be relieved or death would soon supervene.

On the evening of the 16th, I put her upon ergot, as recent discoveries as to its medical properties indicated it in her case. I delayed not; must do something. I soon had her taking tincture ergot, every three hours, in fluid drachm doses. Gave her the first dose at eight o'clock. Remained with her during the night, giving the ergot myself. Imagine my astonishment and joy when, on the morning of July 17th, she looked absolutely like a new being. Could ask for water, say Yes and No, and, better than all, could move her leg and shoulder. Now, Doctor, on this day, July 17th, I began a series of interesting experiments. I could hardly believe my senses, in seeing her so much improved in one short night. Sometimes I would think it simply the vis medicatrix naturæ; again, I would think may be it is the ergot. So I decided to settle the matter. I suspended the ergot at twelve o'clock, on July 17th, and put her back on her old treatment—quinine, iron and strychnine. Had a patient in country, and did not return until six in the evening. I hurried up to see her, and found her laboring under all her bad symptoms, viz: twitching of the muscles, deep coma, etc. I gave her at once a dose of the ergot, and remained with her during the night, giving her the tincture every three hours in fluid drachm doses. By midnight her symptoms were better, and by day-break she looked bright, talked a little, moved her paralyzed limbs, and looked like getting well right away.

July 18th. Well, I am still a "doubting Thomas." Resolved to give her the ergot up to 12 o'clock, and then suspend again, and by night—would you believe it?—every single violent symptom returned. I began its use again, and by morning she was as bright as ever. I no longer doubted the ergot, and resolved to push it; so I gave it to her without cessation, from July 19 to July 23, with a steady improvement. On the 24th, I concluded to stop the remedy, for fear of producing unpleasant effects, but no sooner done than backward tendency ensued. I began its use again, and in a few hours all unpleasantness subsided. The fact is beyond doubt, that she could be oscillated back and forth as a pendulum. I longed to see her recover, but I must confess I did delight in watching with what promptness her case obeyed the drug.

In fact, I was sometimes cruel enough—partly through fear of injurious effects—to suspend its action and let her start backward, and then rush to her rescue with this potent remedy, and in no single instance did it deceive me.

I continued to give the ergot up to August 1st, with no unpleasant effect. She is, to-day, August 15th, sitting up, eating enough, and doing well. Such, Doctor, has been the beginning, middle, and ending, of, to me, the most remarkable and interesting case of my experience.

Yours truly,

W. C. Blalock, M.D.

